# Low frequency transcranial magnetic stimulation of right posterior parietal cortex reduces reaction time to perithreshold low spatial frequency visual stimuli

**DOI:** 10.1038/s41598-020-59662-4

**Published:** 2020-02-21

**Authors:** Seth Elkin-Frankston, Richard J. Rushmore, Antoni Valero-Cabré

**Affiliations:** 1 Laboratory of Cerebral Dynamics, Plasticity and Rehabilitation, Department of Anatomy and Neurobiology, Boston University School of Medicine, Boston, MA United States; 2 Psychiatric Neuroimaging Laboratory, Brigham and Women’s Hospital, Boston, MA United States; 3 Center for Morphometric Analysis, Massachusetts General Hospital, Boston, MA United States; 4 Cerebral Dynamics Plasticity and Rehabilitation Group, FRONTLAB Team ICM & CNRS UMR 7225, INSERM UMR 1127, Sorbone Universtité & LPNC CNRS UMR 5105-TREAT vision, Service de Neurologie, Fondation Ophtalmologique Adolphe de Rothschild, Paris, France; 5 Cognitive Neuroscience and Information Technology Research Program, Open University of Catalonia (UOC), Barcelona, Spain; 6 U.S. Army Combat Capabilities Development Command Soldier Center, Natick, MA United States

**Keywords:** Perception, Sensory processing

## Abstract

Research in humans and animal models suggests that visual responses in early visual cortical areas may be modulated by top-down influences from distant cortical areas, particularly in the frontal and parietal regions. The right posterior parietal cortex is part of a broad cortical network involved in aspects of visual search and attention, but its role in modulating activity in early visual cortical areas is less well understood. This study evaluated the influence of right posterior parietal cortex (PPC) on a direct measure of visual processing in humans. Contrast sensitivity (CS) and detection response times were recorded using a visual detection paradigm to two types of centrally-presented stimuli. Participants were tested on the detection task before, after, and 1 hour after low-frequency repetitive transcranial magnetic stimulation (rTMS) to the right PPC or to the scalp vertex. Low-frequency rTMS to the right PPC did not significantly change measures of contrast sensitivity, but increased the speed at which participants responded to visual stimuli of low spatial frequency. Response times returned to baseline 1-hour after rTMS. These data indicate that low frequency rTMS to the right PPC speeds up aspects of early visual processing, likely due to a disinhibition of the homotopic left posterior parietal cortex.

## Introduction

The posterior parietal cortex (PPC) is considered a higher order cortical region that integrates a diverse range of perceptual information and contributes to guided motor responses, action-planning and decision-making^1–3^. This region is comprised of anatomically and functionally distinct cortical areas including the intraparietal sulcus (IPS), the neighboring angular gyrus and the supramarginal gyrus. The human right PPC and its purported homologue in monkeys have been found to be important components of both the dorsal visual stream^4^ and the dorsal fronto-parietal attention network^5^. Among other functions, these areas contain body/head-centric as well as retinotopic representations^6^ for both personal and peri-personal spaces, which are crucial for visuospatial orienting processes^7–10^.

The PPC exerts considerable influence over activity in primary visual areas^11–13^. The posterior parietal cortex is active prior to or at the same time as primary visual cortex upon stimulus presentation, and responses and activity in early visual areas is enhanced after preceding activation of parietal cortex^8,14–17^. As a result, the posterior parietal cortex is in a position to guide and adjust the emergent visual signals as they enter early visual areas, likely by modulating the input gain of primary visual neurons^13,18–20^. Anatomical studies have shown that parietal regions have strong reciprocal connections with early extrastriate visual areas V2, V3, V3a and V4^6,21–23^. A more recent study has even unveiled a sparse projection from posterior parietal areas to V1^24^.

Much of the information about the influence of parietal cortex on early visual processing has come from anatomical and functional studies in the non-human primate. In the human, transcranial magnetic stimulation (TMS), a technique that non-invasively modulates specific brain areas and their associated networks^25^ has supported these results and provided evidence that extrastriate visual regions and PPC exert top-down control over early visual areas^19,20,26^. More specifically, studies using chronometric designs have demonstrated that visual awareness, in particularly to motion, is dependent upon feedback projections from extrastriate MT/V5 areas to V1^19,20^. Further evidence supporting top-down modulatory control over early visual processing comes from offline rTMS experiments targeting the right IPS. In these studies, visual detection was impaired for targets presented in the visual field contralateral to the stimulated hemisphere^27–29^. Finally, right but not left PPC stimulation produces intensity- and stimulus-dependent changes in BOLD activity in occipital visual areas (MT/V5, V1–V4)^30,31^.

In the present study, we sought to examine the degree to which the PPC can modulate visual performance through top-down interactions on early visual areas. We used a task designed to test basic visual function – contrast sensitivity of a centrally-located Gabor stimulus at 3 cycles per degree (cpd) and at 12 cpd spatial frequencies. Performance on this test was evaluated before and after the delivery of inhibitory 1 Hz repetitive Transcranial Magnetic Stimulation (rTMS) to the right posterior parietal cortex. Low and high spatial frequencies of perithreshold visual stimuli were evaluated separately because evidence suggests that they are processed differently in the cerebral cortex^32–37^ such that lower spatial frequency stimuli are more associated with the magnocellular/dorsal stream, whereas higher spatial frequency is preferentially processed by parvocellular/ventral stream structures. Since a major target of the dorsal visual stream is the posterior parietal cortex^22,38,39^, we hypothesized that top-down influences from the PPC would selectively reduce the contrast sensitivity of the lower but not higher spatial frequency stimuli.

## Results

**Figure 1:**
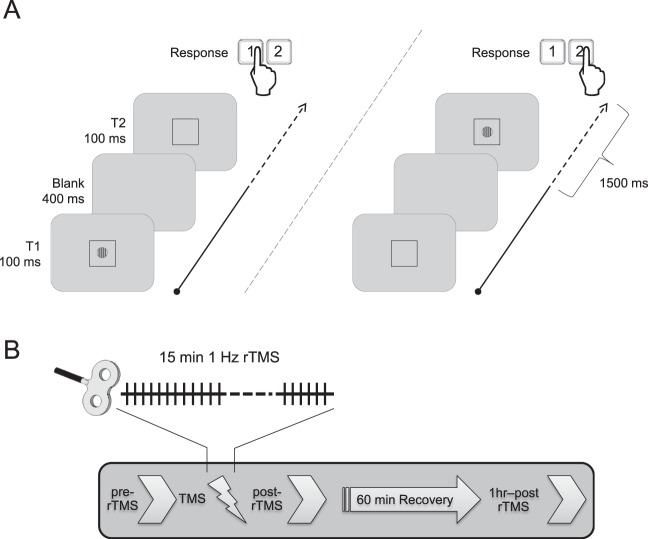
Visual paradigm and study design. (**A**) Trials consisted of two time intervals (T1 & T2) separated by a 400 ms-long period of a blank screen. A Gabor patch could be flashed for 100 ms in either T1 or T2, with equal probability. At the start of the experiment, observers were told to indicate using a button press the interval in which the Gabor patch appeared in within a window of 1500 ms following the presentation of the 2^nd^ interval (T2). (**B**) Contrast sensitivity was measured at three different evaluation points; (1) baseline (pre-rTMS), (2) immediately following the application of 1 Hz rTMS for 15 minutes (post-rTMS), and (3) after a one hour recovery period (1-hour-Post-rTMS).

Behavioral data for contrast sensitivity were obtained using an adaptive staircase method in which contrast sensitivity threshold was estimated based on the average of 20 reversals around threshold values^40^. The procedure was carried out before and following 1 Hz rTMS to the PPC or the vertex (Fig. 1). Baseline contrast sensitivity for visual stimuli at low (3 cpd) and high (12 cpd) spatial frequency were consistent with values found in previously published reports^41–43^. Analysis of contrast sensitivity following rTMS to right PPC or the vertex showed that contrast sensitivity did not vary with stimulation region (PPC, vertex), stimulation time point (pre-rTMS, post-rTMS, 1hr-post-rTMS), or the interaction of these factors (all F < 1.0, all p > 0.05).

**Figure 2:**
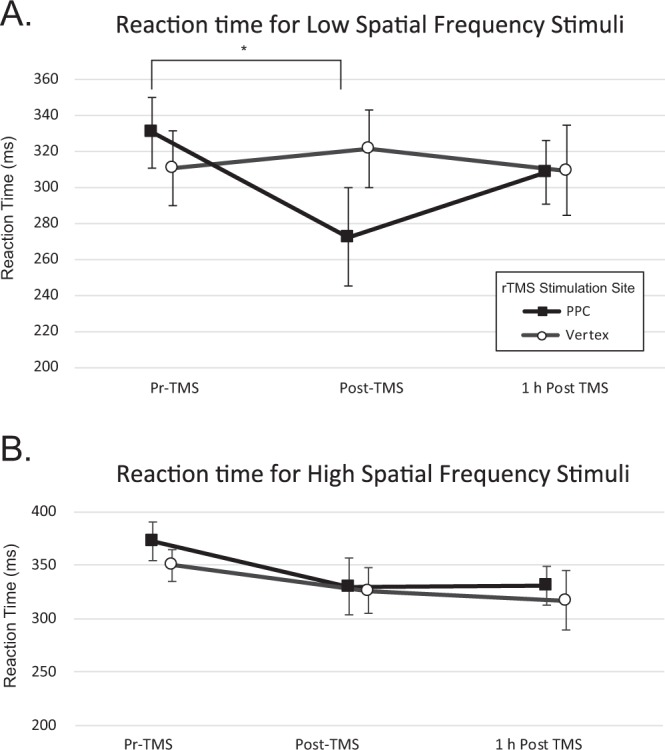
Effect of rTMS to right PPC and vertex on reaction time. Low frequency rTMS to the right posterior parietal cortex (PPC), but not to the scalp vertex, reduced the latency at which participants responded to perithreshold central visual stimuli. This effect was present for low spatial frequency Gabor stimuli (**A**), but not high frequency Gabor stimuli (**B**). No effect was found when the vertex was stimulated. The asterisk indicates a significant difference between pre-TMS and post-TMS reaction time after right PPC stimulation with an adjusted p value of p < 0.016 after Bonferroni correction for multiple comparisons. Error bars represent the standard error of the mean.

The reaction time with which participants responded to visual stimuli was analyzed. Since reaction time analysis was not the main goal of this study, this analysis was exploratory. We used a mixed model with rTMS time point ‘rTMS Time’ (pre-rTMS, post-rTMS, 1hr-post-rTMS) as a repeated measure and stimulation site ‘Stim Site’ (vertex, right PPC) as a main factor. Since the task used sequential presentation of visual stimuli, we also included stimulus presentation order ‘Flash Number’ (T1 or T2) as a factor to test whether reaction times varied depending on the presentation order of the visual stimulus, and if so, whether this order interacted with rTMS time point ‘rTMS Time’ or with rTMS stimulation site ‘Stim site’. Results for 3 cpd visual Gabor stimuli showed a significant effect of rTMS time point (F(2,25) = 3.97, p = 0.03), and a significant effect of stimulus presentation order (F(1,15) = 28.2, p < 0.0001; Table 1). No main effect of stimulus site (F(1,22) = 0.06, p = 0.81) was found, but there was a significant interaction between stimulation site and rTMS time point (F(2,58) = 7.6, p = 0.001). All other interactions were not significantly different (all F < 1.0, all p > 0.05). Results indicate that reaction time varied according to stimulation presentation order irrespective of stimulus site, but the lack of interaction effects show that this contribution was the same regardless of stimulus site or rTMS time point (Table 1). Significant interaction effects between rTMS time point and stimulation site were followed by post-hoc tests to examine reaction time at different time points relative to the rTMS (pre-rTMS, post-rTMS, 1hr-post-rTMS), using Bonferroni correction for 15 comparisons (adjusted p = 0.003). A significant comparison was only observed between pre-rTMS and post-rTMS reaction times (t = 4.39, p < 0.0001). This result shows that reaction times decreased after rTMS to the right PPC, but not vertex (Fig. 2). Analysis of reaction times for 12 cpd visual stimuli showed an effect of rTMS time point (F(2,29) = 4.4, p = 0.02) and stimulus presentation order (F(1,16) = 50.7, p < 0.0001)). Nonetheless, interaction effects did not reach significance, indicating no effect of right PPC rTMS on reaction time for 12 cpd visual stimuli.

**Table 1:** Statistical tables for the effects of rTMS on reaction time values to visual targets (Gabor patches) at spatial frequencies of 3 cycles per degree (cpd) (above) and 12 cpd (below). Factor: Stim Site (PPC, Vertex); rTMS Time (Pre-rTMS, post-rTMS), 1h-post-rTMS), Flash Number (T1, T2).

Source	DF Num	DF Den	F Ratio	Prob > F
**3 cpd Gabor Patches **				
Stim Site	1	21.8	0.059024	0.8103
rTMS Time	2	25.5	3.9794925	0.0313
Stim Site*rTMS Time	2	58.2	7.6194111	0.0012
Flash Numb	1	15.1	28.243444	<0.0001
Stim Site*Flash Number	1	38.9	0.0005819	0.9809
rTMS Time *FlashNumber	2	29.0	1.3974635	0.2634
Stim Site*rTMS Time *Flash Number	2	29.0	2.7664654	0.0795
**12 cpd Gabor Patches**				
Stim Site	1	12.6	0.4458494	0.5164
rTMS Time	2	28.6	4.4208761	0.0212
Stim Site*rTMS Time	2	63.2	0.5520175	0.5785
Flash Number	1	16.3	50.751529	<0.0001
Stim Site*Flash Number	1	33.7	0.0005266	0.9818
rTMS Time *Flash Number	2	39.2	2.9374599	0.0648
Stim Site*rTMS Time *Flash Number	2	39.2	0.3192382	0.7286

Finally, equivalence was tested on normalized data (post-rTMS/pre-rTMS) for contrast sensitivity and reaction time data at both 3 cpd and 12 cpd Gabor targets. These comparisons revealed statistical equivalence between contrast sensitivity after rTMS stimulation to the right PPC and the scalp vertex sites (with a d = 0.3) for 12 cpd and 3 cpd Gabor patches, indicating that there was no statistical difference between these two groups. Similarly, reaction time data for 12 cpd targets showed statistical equivalence between vertex and PPC stimulation sites. Equivalence testing of reaction time data for 3 cpd Gabor targets showed statistical significance, but not statistical equivalence for PPC and stimulation groups.

## Discussion

The goal of this study was to determine the role of the right PPC in the top-down modulation of a visual behavior linked to early visual cortical processing. Contrast sensitivity was evaluated because of its frequent use in the study of low level visual capabilities^43–47^. Moreover, contrast sensitivity as well as other similar measures have been shown to be modifiable by training, allocation of attention resources, and the external modulation of activity in higher cortical brain areas^30,31,40,45,48,49^. Many of the modulatory effects on contrast sensitivity have traditionally been interpreted as the ability of attentional or other systems to increase visual saliency or effective contrast through top-down control by adjusting either contrast or response gain mechanisms^50–54^. The posterior parietal cortex (PPC) is a key region in the attentional system, which exerts influence over activity in primary and extrastriate cortex by gating and/or selectively modulating neuronal activity^51,55–61^. Previous research has shown that PPC stimulation modulates activity in early visual areas^30,31,62–64^ and has provided strong evidence that short bursts of rTMS to the right, but not the left PPC modulates blood-oxygen level dependent (BOLD) activity in both left and right early visual areas regardless of visual target eccentricity^31^. Based on this evidence, we hypothesized that the suppression of the PPC would degrade contrast sensitivity. Our results did not confirm this hypothesis. Low frequency rTMS to the right PPC did not produce any immediate or long-term effect on contrast sensitivity for visual stimuli of either spatial frequency. This outcome suggests that the right PPC does not modulate contrast sensitivity for the spatial frequency stimuli used in this study, at least under the conditions of this experiment.

The right PPC has been primarily implicated in the allocation and redirection of attention, particularly to visual stimuli appearing at peripheral eccentricities in the contralateral visual field^65^. Although research has shown that PPC exerts effects on the activity in early visual areas irrespective of eccentricity^31^, it is reasonable to surmise that any top-down effects may be specifically tied to the detection of peripherally placed stimuli with high salience and contrast that would cause a redirection of attention or a reorienting of gaze. Since perithreshold visual stimuli similar to those employed in the present study are unlikely to precipitate a quick redirection of gaze, they may have been unable to warrant either prompt activation of the right PPC or top-down modulation of contrast in early visual areas. Alternatively, our use of a relatively low intensity stimulus may not have sufficiently modulated the activity of the right PPC modulatd with 1 Hz rTMS.

To gain further insight into a possible relationship between the right PPC and perithreshold stimuli, we evaluated reaction time datasets using a follow-up analysis. Reaction times to low, but not high spatial frequency perithreshold visual stimuli were faster immediately after TMS, suggesting that the suppression of right PPC area facilitated processing speed for low spatial frequency stimuli. But how might deactivation of the right PPC produce an increase in processing speed? Paradoxical facilitation following the application of suppressive rTMS patterns has a long precedence in the non invasive brain stimulation literature^27,28,66–68^. This type of facilitation is known to be particularly prevalent when stimulating regions of the right PPC in both online^66^ and offline paradigms^27,28^ or in cases in which TMS is applied to other cortical sites including the primary visual cortex^69^. In this instance, the reduction in reaction times may be a consequence of modified interhemispheric interactions^27,67,68^. The two parietal cortices are linked by transcallosal projections and are thought to operate in a state of balance such that when one posterior parietal cortex is active, the other is rendered relatively silent^70–73^. This interaction is mediated by connections that functionally inhibit each other, presumably through transcallosal activation of an inhibitory circuit^74^. Exogenous suppression of the right PPC thus disinhibits the left PPC, and may facilitate functions associated with the left PPC. Such an interaction would explain how a decrease in excitability leads to a reduction in reaction time. The right PPC is involved in attentional mechanisms but appears to be particularly important for the allocation of spatial attention and the detection of high salience visual stimuli^75^. The left PPC, on the other hand, responds well to visual stimuli of low salience, as used in this study. Thus, an increase in processing speed to low spatial frequency perithreshold visual stimuli is consistent with a disinhibition of the left PPC.

These results may have implications for the treatment of conditions in which vision is compromised. Results show that processing speed for perithreshold visual stimuli can be increased by rPPC rTMS without concomitant loss of function for contrast sensitivity. In the case of this study, the increase was temporary, and more work would have to be performed to determine whether multiple sessions of rTMS provide increasing, or lasting benefit, as found using neurostimulation techniques in other cortical regions^76–79^. In addition, research would need to evaluate whether multiple periodical rTMS sessions delivered to the right PPC would reduce behaviors tied to nominal right PPC function, including attentional deployment, visual search, visual orienting and detection^27,28,67,80,81^.

The findings in this paper linking suppression of right PPC with effects on low spatial frequency stimuli may indicate that the modulation of right PPC activity may have an effect on the magnocellular visual stream. As a result, these findings may have implications for novel uses of rTMS in disorders characterized by selective dysfunction in visual subsystems. Visual dysfunctions in schizophrenia has been hypothesized to be a result of problems in the magnocellular visual stream^82^ and recent data have suggested that visual impairments in autism may be characterized, at least in part, by excessive processing of the peripheral visual field, which may come at the cost of processing stimuli at central visual field locations^83,84^. In this context, right PPC deactivation by low frequency rTMS may be indicated as a potential route to bias processing resources towards central visual field representations of early visual areas without unduly affecting basic visual function.

## Materials and Methods

A group of 36 healthy human participants (24 female, 12 male, mean age 24 years) were recruited for this study (Table 2) and were compensated for their participation. All participants were pre-screened prior to admission into the study against a preclusionary criteria of personal or family history of epileptiform disorders, metallic implants, and neuroleptic medications^85^. Informed consent was obtained from all participants. Consenting participants reported no history of neurological or psychiatric disorders and had normal or corrected-to-normal vision. The research protocol implemented in the present study was approved by the Boston University School of Medicine Institutional Review Board and was carried out in accordance with the Code of Ethics of the World Medical Association.

**Table 2:** Demographic information of the experimental groups (9 participants per group) that took part in the current study. Independent groups of participants completed visual performance experiments involving the stimulation of the right PPC or the scalp vertex. Visual detection tasks were carried out in separate experiments with low (3 cpd) or high (12 cpd) spatial frequency Gabors.

rTMS conditions	n	Male ♂	Female ♀	Mean age (years)	Age range (years)
right PPC 3 cpd	9	4	5	23.4	20–27
right PPC 12 cpd	9	2	7	23.7	22–28
Vertex 3 cpd	9	2	7	23.3	20–28
Vertex 12 cpd	9	3	6	24.1	20–28

### Visual paradigm

Briefly, contrast sensitivity was measured with a standard two alternative forced choice paradigm (2AFC) task using single vertically-oriented circular Gabor stimuli subtending 3° of visual angle^40,48^. programmed in-house with C# using Microsoft’s Visual Studio 2010 (Microsoft, Redmond, WA, USA). Participants observed stimuli with their eyes’ *canthi* positioned 57 cm from the computer monitor so that 1° of visual angle measured 1 cm along the horizontal axis of the computer monitor. Each trial consisted of two sequentially presented visual stimuli, presented for 100 ms and separated by 400 ms. Stimuli consisted of a centrally located black border (subtending 13° of visual angle (400 × 400 pixel grid, border width = 1 pixel). Gabor stimuli were flashed for 100 ms with equal likelihood during either the first (T1) or the second (T2) time interval. Participants were instructed to respond as quickly and accurately as possible signaling whether the Gabor appeared in either the first (T1) or the second (T2) interval using the keys “1” for T1 and “2” for T2 on a numeric keypad with the index and middle fingers of the right hand. RT was calculated as the duration between the end of the second interval (T2) and the time at which a finger response was executed (Fig. 1A). When participants were unsure which interval contained the stimuli, they were encouraged to make their best guess. Participants were given 1500 ms to provide an answer before the next trial was automatically started. In the case of an absent response an audible warning indicated an error and the trial was repeated using the same contrast value.

Contrast sensitivity measures were obtained using an adaptive staircase procedure). Gabor stimuli were initially presented at 5.0% Michelson contrast: C = (L_max_ − L_min_)/(L_max_ + L_min_). Stimulus contrast was subsequently increased or decreased according to task performance. The first two reversals used a 1-down/1-up rule, i.e., Gabor contrast was either increased or decreased a step value of 0.6% Michelson contrast in response to an incorrect or a correct response, respectively. At the initiation of the third reversal a 3-down/1-up rule was implemented with a step value of 0.2%; i.e., following 3 correct responses, contrast value was decreased by 0.2% and following each incorrect response it was incremented by 0.2%. Trials were continually presented until twenty reversals were completed following the 3-down/1-up rule with a step value of 0.2% contrast^40^. A reversal was counted when there was a shift of sign between contrast values for the current and the just previously presented trial. Individual contrast threshold values were calculated from the final twenty reversal values from each evaluation time point. Contrast sensitivity was then obtained by calculating the inverse of the contrast threshold; CS = 1/C (reported in log units). Reaction time values for all correct responses completed during the 3-down/1-up rule were included in our analyses. Reaction time values included in the analysis were restricted to responses between 50 ms and 1000 ms^86^.

### Transcranial magnetic stimulation

Repetitive TMS was performed using an air-cooled 70 mm figure-eight coil attached to TMS machine (Superapid^2^, Magstim, Withland, UK) and fixed in position with the aid of a ‘magic-arm’ (Manfrotto Bassano del Grappa, Italy). Patterns of 1 Hz rTMS were delivered to the middle portion of the right PPC, as well as to an additional control location (scalp vertex, Cz) for a total of 15 minutes (900 pulses total). Low-frequency rTMS was chosen due to its minimal seizure risk and because similar rTMS interventions to parietal and occipital locations have been used to investigate visual perception^27,28,69^. Stimulation intensity was initially set up at 80% of each participant’s motor threshold and occasionally, adjusted for individual comfort. The final stimulation (± Standard Deviation) intensities applied to participants averaged 62 ± 10% of the maximum rTMS machine output.

As done elsewhere^27,28,69^, for the right PPC stimulation condition, the TMS coil was placed over a scalp location corresponding to site P4 of the 10–20 EEG system and the middle portion of the IPS overlaying the right Angular Gyrus (AG)^87^. The site of vertex stimulation was located at the intersection between a line connecting the left and right preauricular points and the nasion-inion line (Cz on the international 10–20 EEG system). The correct locations of our targets for the PPC and vertex sites were verified using individual structural MRI images with vitamin E high contrast markers placed on the targeted regions at the P4 site and Cz sites indicated above. To this end, high-resolution T1-weighted images were acquired on a 3 Tesla Philips MRI scanner using a 3D-turbo field echo (TFE) T1-weighted sequence (equivalent to MP-RAGE). Parameters included a field of view (FOV) 240 mm (RL) × 256 mm (VD) × 192 mm (AP); Fold-over-axis: RL, data matrix: 160 × 160 × 144 zero-filled to 256 in all directions (approx 1 mm iso-voxel native data), TR/TE = 9 ms/6 ms, flip angle = 8°. The pial surface of the brain was visualized using FSL’s^88,89^ Brain Extraction Tool^90^ then superimposed on a whole head image. As expected^87^, in all cases the vitamin E marker was located above the level of the middle portion of the right IPS and 0.5–0.7 cm ventral to the sulcus in the lateral bank corresponding to the angular gyrus. In this location, the coil was oriented in a lateral-to-medial and caudal-to-rostral orientation at approximately 45° from the interhemispheric longitudinal midline.

Detailed inspection also confirmed that vitamin E capsules placed over the vertex location overlaid the longitudinal interhemispheric fissure, at which point the midsagittal and interaural lines intersect. This area closely corresponds to the medial origin of the postcentral gyrus^83^. The TMS coil was oriented in a lateral-to-medial and caudal-to-rostral orientation.

### Experimental sessions

Participants were assigned to two independent groups, each associated with one of two stimulation conditions: An experimental condition receiving rTMS to the right PPC and a control condition receiving rTMS to the scalp vertex. For the right PPC rTMS and vertex rTMS conditions, participants were randomly assigned to one of two groups (n = 9 participants each). One group was tested with high spatial frequency (12 cpd) stimuli while the other was tested using low spatial frequency (3 cpd) achromatic Gabor stimuli. Each experimental session required approximately 120 minutes (including preparation, performance measurements and stimulation time). Participants were first fitted with a tight fitting Lycra swim cap. The location of the targeted region of cortex was determined and the site of stimulation and coil orientation marked. At the start of each experiment, a baseline visual performance measure was obtained (pre-rTMS). Repetitive TMS was then applied for 15 minutes at 1 Hz. Immediately following rTMS, visual performance was re-measured (post-rTMS). After a one hour ‘washout’ period, contrast sensitivity was obtained (1h-post-rTMS) for the last time during the session (Fig. 1B). Prior to the baseline (pre-TMS) time point, participants completed a practice performance task. The purpose of this practice session was to limit immediate learning effects and to ensure that participants fully understood the task. This practice condition was not included in the analyses.

### Statistical analysis and data presentation

Data were analyzed using JMP 13.0 (SAS, Cary, NC USA). Contrast sensitivity values and reaction times were evaluated using linear mixed models, which accounts for the inter-subject variability in the repeated measure by crossing random effects with independent variables. These models were fit for restricted maximum likelihood. Performance to low and high spatial frequency Gabor stimuli were analyzed separately. For the reaction time analysis, we added an additional factor which was whether the stimulus was flashed in the first (T1) or the second (T2) 100 ms interval and tested the possibility of an order effect interacting with stimulation site (PPC, vertex) or TMS time point (pre-rTMS, post-rTMS, 1h-post-rTMS). Significant main effects were followed up with planned two-tailed Student’s t-tests, and Bonferroni correction was used to modify the p value when multiple comparisons were used. Significance was set at p = 0.05.
